# Aptamer-mediated delivery of therapeutic oligonucleotides in glioblastoma

**DOI:** 10.1016/j.tranon.2025.102485

**Published:** 2025-07-26

**Authors:** Giorgia Castellani, Mariachiara Buccarelli, Enza Cece, Martina Offi, Lucia Ricci-Vitiani

**Affiliations:** a Department of Oncology and Molecular Medicine, Istituto Superiore di Sanità, Rome, Italy; b National Centre for Innovative Technologies in Public Health, Istituto Superiore di Sanità, Rome, Italy; c Department of Neuroscience, Catholic University School of Medicine, Rome, Italy

**Keywords:** Aptamers, Nucleic acid therapeutics, Glioblastoma, Delivery system, Nanoparticles

## Abstract

Aptamers are single-stranded oligonucleotides with great versatility, acting as therapeutic molecules with anticancer properties but also as delivery systems. A growing number of studies showed that aptamers can be properly designed to recognize a specific target, to conjugate drugs, nanoparticles or nucleic acid therapeutics (NATs) and to cross the blood brain barrier. In this review, we summarized the main advantages of aptamers as delivery system, focusing our attention on aptamer-mediated delivery of therapeutic oligonucleotides in glioblastoma. Glioblastoma represents the most common and aggressive primary malignant brain tumor in adults, with a median survival ranging from 14.6 to 20.5 months. Currently, the standard treatment of newly diagnosed tumor includes surgical resection followed by radiotherapy and chemotherapy with the alkylating agent temozolomide. However, the overall efficacy is limited and most patients relapse within a few months, calling attention on the development of alternative therapeutic strategies. Particularly, we discussed research studies on NAT delivery mediated by aptamer chimeras and aptamer-nanoparticle complexes able to impair various processes involved in glioblastoma tumorigenesis. This overview will help to highlight how aptamers, thanks to their advantages, could represent a promising tool for the development of innovative therapeutic approaches for glioblastoma treatment.

## Highlights

• NATs are able to precisely target cancer-related genes and “undruggable” pathways.
• Aptamers are valuable delivery systems thanks to specific targeting ability and high versatility.
• Aptamers release their cargo into GBM cells through their ability to cross the blood-brain barrier.

## Graphical abstract

**Figure:**
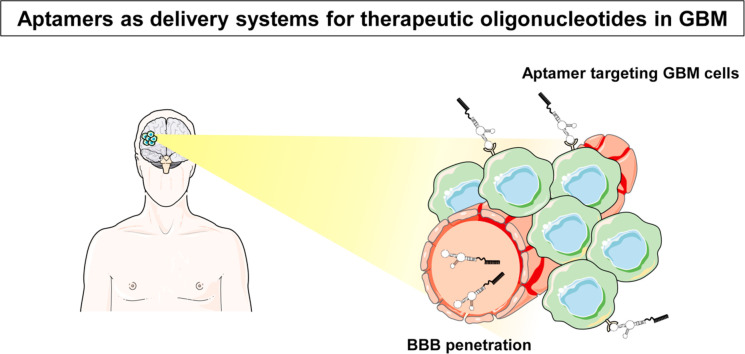

## Background

Glioblastoma (GBM) is the most frequent form of primary intracranial tumor in adults, accounting for 50.1 % of all malignant primary central nervous system (CNS) tumors [1]. According to the most recent classification (fifth edition) of CNS tumors, developed by the World Health Organization (WHO), GBM represents the most lethal form of *isocitrate dehydrogenase* (*IDH*)-wild type diffuse adult-type astrocytoma, where the methylation status of the promoter of *O^6^-methylguanine-DNA methyltransferase* (*MGMT*) gene is a prognostic factor [2]. The standard therapeutic regimen for GBM managing consists on maximal safe surgical excision, followed by radiation plus concomitant and maintenance chemotherapy with temozolomide (TMZ) [3]. Despite the addition of tumor-treating fields therapy to maintenance TMZ, there are no significant improvements in prolonging GBM patient survival rates that currently is ranging from 14.6 to 20.5 months. Frequently, GBM patients develop relapse within a period of 6–9 months, and available second-line treatments do not show encouraging results [[4], [5], [6], [7], [8]]. Indeed, although the recent inclusion of the multikinase inhibitor regorafenib as a preferred regimen in relapsed GBM treatment in several countries, patients’ response is variable and not prolonged [[9], [10], [11]]. Therefore, due to the rapid evolving clinical course that commonly characterizes GBM, there is an urgent need to develop more effective novel therapies.

Currently, several factors limit the efficiency of GBM standard therapy including, complex alterations in tumor driving genes that causes a wide inter- and intra-tumor heterogeneity; inadequate drug or agent delivery across the blood-brain barrier (BBB); lack of drugs able to specifically target only the tumor component, without undesired effects affecting non-malignant cells [12,13].

Nucleic acid therapeutics (NATs) have received particular attention in the last decade, since they enable precise regulation of the expression of cancer-related genes, representing a valuable technology for precision oncology [14]. In this context, advances in the comprehension of GBM molecular biology and genetic allowed the identification of the main alterations in cancer-related genes, including *PDGFR, TP53, TERT* and *EGFR*, and the involvement of aberrant networks regulated by non-coding RNAs (ncRNAs) [15,16]. Among ncRNAs, microRNAs (miRNAs) have been identified as critical modulators of GBM tumorigenesis, radio- and chemo-resistance, stem cell maintenance, epigenetic regulation, tumor angiogenesis, and invasion [16]. Therefore, targeting deregulated miRNAs, taking advantage of synthetic anti-microRNAs (anti-miRs), antisense oligonucleotides (ASOs) or miRNA mimics, represents one of the promising NAT-based therapeutic approaches [17]. Over the years, a growing numbers of NAT-based strategies have been developed, aimed at selectively activating or silencing specific tumor driving genes and the underlying pathways that are considered “undruggable” by standard therapeutic approach. Of note, several NAT-based therapy have been tested in clinical trials as promising method for GBM management [[18], [19], [20], [21], [22]]. Although the potential of this approach, the delivery of NATs to specific tissues and their cellular uptake represent important challenges and drawbacks [22]. Aptamers, a class of single stranded RNA or DNA oligonucleotides folded in a unique three-dimensional (3D) structure, have emerged as an interesting class of carriers thanks to their capacity to recognize their ligands with high affinity [23]. Particularly, aptamers can selectively drive NATs in a cell- or tissue-specific manner, improving their efficacy and biodistribution in the tissue of interest and minimizing the possible off-target effects due to the accumulation in non-target tissues. For aptamer-mediated delivery of NATs, two different strategies can be distinguished: i) aptamers can be directly conjugated with NAT; ii) aptamers can be combined with nanoparticles (NPs), forming aptamer-nanoparticle (Ap-NP) complexes, and NAT is loaded inside. In this review, we provide a comprehensive overview of the most interesting examples of aptamer-mediated delivery of NATs in GBM and summarize the main advantages of aptamers as delivery system.

### RNA-based therapeutic approaches

Increasing studies have reported that modulation of expression of specific genes encoding cancer-related proteins through NATs may represent a promising strategy for GBM treatment [17]. NATs are different forms of DNA or RNA, ranging from few to several thousand of nucleotides, which represent valuable tools for precision medicine and long-term treatment. They are able to target selectively cancer-related genes and “undruggable” pathways compared to conventional treatments. Based on the mode of action, NATs can be classified in ASOs, anti-miRs, miRNA mimics, small interfering RNA (siRNAs), CRISPR-Cas guide RNAs (gRNAs), catalytic nucleic acids (CNAs) and aptamers [17,24].

ASOs are single-stranded DNA sequences that bind by Watson-Crick base pairing with specific target messenger RNA (mRNA). These molecules can inhibit the translation of target mRNA through three different mechanisms: ribosome blockage by steric hindrance, mRNA degradation through RNAse H1 or splicing modulation [22,25,26]. Moreover, ASOs designed to be fully or partially complementary to miRNAs are called anti-miRs and inhibit the interaction between the miRNA of interest and its target mRNAs [27]. MiRNA mimic is a class of single/double strand synthetic RNA molecules that exploit the function of the endogenous miRNA [28]. SiRNAs differ from ASOs, because are double-stranded oligonucleotides that recognize their complementary target mRNA by perfect pairing and triggering its degradation through the RNA-induced silencing complex (RISC) [29,30]. These types of NATs permit only the silencing of target genes, whereas the CRISPR-Cas system allows genetic manipulation through deletion, insertion, or modification of DNA or RNA with single nucleotide precision using a gRNA [31]. Although it has remarkable potential in restoring or silencing the expression of cancer-related genes, the safety and ethical problems of this technology are still debate [32,33]. Another interesting NAT system is represented by CNAs, molecules of RNA (ribozyme) or DNA (DNAzyme) with biological catalytic function. CNAs can catalyse several type of reactions, including RNA cleavage, promoting thus gene silencing with an RNA interference (RNAi)-independent mechanism [34,35]. Aptamers, also known as “chemical antibodies”, are single-stranded oligonucleotides that can interact to their specific targets thanks to their three-dimensional (3D) structure [23].

**Fig. 1:**
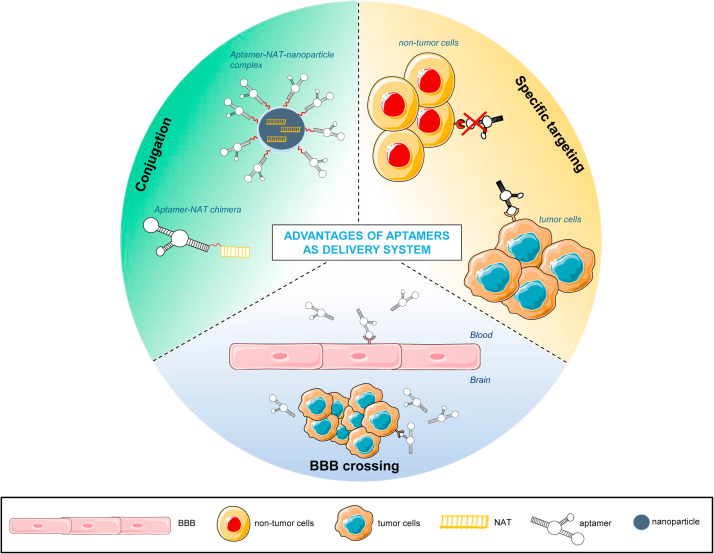
**Advantages of aptamers as delivery system.** Schematic representation of advantages of aptamers as delivery system in GBM treatment. Parts of the figure are drawn using pictures from Servier Medical Art (https://smart.servier.com (accessed on 17 February 2025)).

The field of NATs has become an outstanding area of anti-cancer therapy research and has advanced considerable in last years. However, their susceptibility for degradation associated to nucleases and their low permeability to cross the cell membrane due to their phosphate backbone with negative charge, pose a challenge for their clinical application. To overcome these limitations, several chemical modifications are introduced in the backbone, base and sugar, of NATs to protect them from degradation. These modifications include: backbone modifications such as phosphorothioate (PS) or phosphorodiamidate morpholino oligomer (PMO); sugar modifications at the 2′ position most commonly, such as 2′-O-methylation (2′-O-Me), introduction of 2′-O-methoxyethyl group (2′-MOE), and 2′-fluorination (2′-F); base and sugar modifications such as 2′-MOE-5-Me-cytidine and N1-Me-pseudouridine; [36]. An increasing number of delivery systems has been developed to enhance cell internalization, that can be distinguished in viral vector-based (*e.g.*, adenovirus vectors, adeno-associated virus vectors, and lentivirus vectors) and nonviral-based (*e.g.*, DNA nanostructures, inorganic NPs and lipid NPs) delivery systems [24,37]. Aptamers belong to the nonviral-based delivery systems [38]. Unlike other NATs, aptamers have a great versatility, acting as therapeutic molecules with anticancer properties in GBM cells but also as delivery systems for drugs, NPs and other types of NATs [39]. The main advantages of aptamers as delivery system are summarized in Fig. 1.

### Aptamers for the delivery of therapeutic oligonucleotides

Aptamers are synthetic single-stranded DNA or RNA oligonucleotides that are folded in 3D-structures composed by hairpins, loops, bulges, kissing stem-loops or G-quadruplex constructors [23]. Thanks to their 3D conformation, aptamers can finely recognize the difference between a target and non-target molecule and are able to bind a wide range of targets such as proteins, oligonucleotides, toxins, small inorganic compounds, peptides and cells with strong specificity [40].

To develop aptamers against a specific target, an *in vitro* selection process called Systematic Evolution of Ligands by EXponential enrichment (SELEX) is used. The traditional SELEX method can be summarized in three steps: selection, partitioning, and amplification. Before the selection steps, a nucleic acid library is generated, which consists of 10^14^–10^15^ different unique sequences (20–50 nucleotides). During the selection step, the library is incubated with purified target of interest for a specific time. In the partitioning step, the unbound sequences are separated from the target-bound sequences, and removed. In the last step, the target-bound sequences are eluted from the target and amplified by PCR (DNA SELEX) or reverse transcribed to cDNA and subsequently transcribed to RNA using T7 RNA polymerase (RNA SELEX). After that, the PCR amplification products can be utilized for the following round of selections, approximately for 6–18 rounds in order to obtain aptamers with the highest affinity to the target of interest [41]. However, this traditional method can be time-consuming, often taking weeks to months to find potential aptamer candidates, and the rate of success is moderately low. With the introduction of high-throughput technologies such as microfluidic chip technology, capillary electrophoresis and high-throughput sequencing method, SELEX process has evolved and different variants (M-SELEX, CE-SELEX and HT-SELEX) have been developed to optimize the selection and the screening of aptamers [[42], [43], [44]]. Furthermore, the development of the cell-SELEX and the whole-organism *in vivo* SELEX has allowed the generation of aptamers capable of distinguishing between tumor cells and non-tumor cells and of crossing the BBB. Particularly, the incubation of the nucleic acid library with living cells - used as “targets”- (cell-SELEX) offers a solution to identify aptamers able to target only tumor cells reducing the toxicity and undesired effects on the healthy cells [45]. An increasing numbers of DNA aptamers, including GBI-10, GMT8, WYZ-41a, WYZ-50a, W5–7, S6–1b, and RNA aptamers as Gint4.T, 40 L, A40s able to selectively target GBM cells have been developed [[46], [47], [48], [49], [50], [51], [52], [53]]. Intriguingly, some of them such as, the aptamers 40 L, A40s and W5–7 specifically recognize a rare population of GBM cells, namely GBM stem-like cells (GSCs), contributing to the resistance to conventional GBM therapies [51,52]. The whole-organism *in vivo* SELEX represents a powerful method for developing aptamers able to cross the BBB and penetrate brain tissues [54]. Taking advantage of this SELEX process, Cheng and colleagues identified the RNA aptamer A15 that was able to bind to brain capillary endothelia and to penetrate into the brain parenchyma [54]. An alternative strategy to design aptamers able to cross the BBB is the identification of aptamers that target transmembrane proteins located on the BBB. An example is the bi-functional DNA aptamer TEPP that binds to both transferrin receptor (TfR) located on BBB endothelia cells and to the epithelial cell adhesion molecule (EpCAM) located on tumor cells within the brain. Thanks to its unique structure, TEPP is capable of transcytosing the BBB and selectively targeting EpCAM^+^ brain metastases [55]. In support of the previous work, several studies have demonstrated that conjugating aptamers against TfR with chemotherapeutic agents or NPs is a good strategy to enhance the BBB penetration in GBM [56,57].

Herein, we report the studies involving aptamer-mediated delivery of NATs in GBM. Their main features are summarized in Table 1. Particularly, for NAT delivery, aptamers can be employed in the following ways to aid targeting: linked by a connector, forming a chimera, or combined with NPs.

**Table 1:** Summary of aptamer conjugates employed as delivery system of NATs in GBM.

**Type of conjugation**	**Aptamer name**	**Aptamer target**	**Nude nanoparticle**	**Outcome**	**Crossing BBB**	**References**
Aptamer-siRNA chimera	Apt-αvβ3	EEF2	not applicable	inhibition of proliferation and induction of apoptosis in U87MG cells	no data available	[58]
Aptamer-siRNA chimera	BA	c-MET	not applicable	inhibition of proliferation and induction of apoptosis in U87MG cells	no data available	[59]
Aptamer-siRNA chimera	PDR3	STAT3	not applicable	inhibition of cell viability in U251-MG	no data available	[60]
Aptamer-siRNA chimera	Gint4.T	STAT3	not applicable	impairment of GBM survival and migration *in vitro*; inhibition of tumor growth and neovascularization *in vivo*	Yes	[61,62]
Aptamer-siRNA chimera-nanoparticle complex	Gint4.T	siHDGF	Mesoporous silica NPs	inhibition of U87MG cell proliferation, migration and invasion; inhibition of tumor growth *in vivo*	no data available	[63]
Aptamer-anti-miR chimera	GL21.T Gint4.T	miR-222	not applicable	inhibition of migration ability and increase of sensitivity to TMZ in U87MG; inhibition of tumor growth *in vivo*	no data available	[64]
Aptamer-anti-miR chimera	GL21.T	miR-222 miR-10b	not applicable	reduction of the intracellular levels of the correspondent miR; increase of the specific target protein levels in U87MG	Yes (GL21.T-anti-miR-10b)	[64]
Aptamer-anti-miR chimera	GL21.T Gint4.T	miR-10b	not applicable	inhibition of tumorsphere formation and migration ability in U87MG and GSCs (in combination with aptamer-miR-137 chimera or Gint4.T-STAT3 chimera)	Yes	[62,65]
Aptamer-anti-miR chimera	A40s	miR-10b	not applicable	reduction of miR-10b levels and increase of protein target expression in GSCs	no data available	[51]
Aptamer-miR chimera	GL21.T Gint4.T	miR-137	not applicable	inhibition of tumorsphere formation and migration ability in U87MG and patient-derived GSCs (in combination with aptamer-anti-miR-10b chimera)	Yes	[65]
Aptamer-miR chimera	A40s	miR-34c	not applicable	increase of miR-34c expression in GSCs	no data available	[51]
Aptamer-antimiRzyme chimera	TfR-aptamer	miR-21	not applicable	inhibition of the expression of endogenous miR-21 in U87MG	no data available	[66]
Aptamer- nanoparticle complex	A15	CD133	liposome	targeting CD133^+^ cells, inhibition of CD133^+^ cell growth and reduction of tumor size in tumor-bearing mice	no data available	[67]
Aptamer-nanoparticle complex	AS1411	nucleolin	DNA tetrahedron	decrease of survivin expression level and induction of GBM cell apoptosis	no data available	[68]
Aptamer-nanoparticle complex	TfR-aptamer	TfR	hollow MnO_2_	silencing TG2 overexpression, inducing GBM cell apoptosis and inhibiting tumor growth in mice	Yes	[57]
Aptamer-nanoparticle complex	SL1	c-Met	liposome encapsulating mesoporous silica NPs	Inhibition of proliferation, migration and invasion *in vitro* and inhibition of tumor growth in subcutaneous xenograft GBM models	no data available	[69]

### Aptamer-siRNA chimeras

Aptamer-siRNA chimeras (AsiCs) have been widely explored in the last years as promising tool for therapeutic siRNA delivery. These constructs are created by different conjugation strategies, such as covalent linkage or base pair annealing by using a universal “sticky-bridge” linker [70]. After internalization into the target cell upon endocytosis, the siRNA portion can be recognized by RNAi machinery, resulting in target mRNA degradation [71].

About 10 years ago, Hussain and colleagues reported for the first time that an RNA aptamer binding specifically to integrin alpha V and integrin beta 3 (αvβ3) can be used to deliver a cytotoxic siRNA to antigen-positive tumor cells. In particular, the authors joined this aptamer to a siRNA that targets eukaryotic elongation factor 2 (EEF2), thus showing its effects in inhibiting cell proliferation and triggering apoptosis *in vitro* in U87MG glioma cell line [58]. The same cytotoxic effects were observed by Zhang and colleagues after DNA aptamer-mediated c-Met siRNA delivery into U87MG-EGFRvIII overexpressing cells [59]. Recently, two studies investigated the aptamer-mediated delivery of siRNA targeting STAT3. In the study of Yoon et al., the delivery of the STAT3-siRNA was achieved by conjugation to the PDR3 RNA aptamer, which is able to recognize PDGFRα. The PDR3-siSTAT3 chimera showed the ability to downregulate STAT3 expression and decrease viability *in vitro* in U251-MG glioma cell line [60]. In the second study, the authors used the Gint4.T aptamer, which specifically binds PDGFRβ that is overexpressed on the surface of GBM cells and associated vascular endothelium, giving GBM targeting ability. Gint4.T was conjugated to STAT3-specific siRNA to develop an AsiC for its delivery. They demonstrated the effects of the aptamer-conjugate in impairing GBM survival and migration *in vitro*, and tumor growth and neovascularization in a xenograft model of GBM [61]. Since PDGFRβ is preferentially associated with the self-renewing GSCs and STAT3 exerts a key role in GSC propagation, the same authors demonstrated that Gint4.T-STAT3 AsiC was also able to impair GSC migration and survival *in vitro* [62]. Interestingly, they showed in a previous study that Gint4.T is able to cross an *in vitro* BBB tri-culture model [65]. The same Gint4.T aptamer was conjugated to a siRNA targeting HDGF, generating an AsiC to endow a nanovehicle with GBM cell-specific binding ability. The authors reported that *in vitro* treatment with the nanovehicle inhibits GBM cell proliferation, migration and invasion. Moreover, the same approach *in vivo* significantly inhibits tumor growth and improve survival in GBM-bearing mice, supporting that this delivery system of nanovehicle with AsiCs could represent a new strategy for efficient GBM treatment [63].

### Aptamer-based miRNA targeting

Various miRNA modulating strategies have been reported in recent years, either as inhibitory or restorative therapies, showing a potential applicability to pathologies with underlying miRNA deregulation. For the effective delivery to target cells, aptamers have been developed as carriers for both anti-miR sequences and miRNA mimics, by means of appropriate conjugation modalities.

In 2015, the study of Catuogno and colleagues provided the first demonstration of an effective and flexible two-component aptamer-based approach for the specific delivery of therapeutic anti-miR sequences. They designed a two-component conjugate with the anti-miR-222 cargo and the GL21.T or Gint4.T RNA aptamer as a delivery carrier, by adopting the stick-based approach to avoid possible interference of the anti-miR sequence on the aptamer proper folding. They reported that treatment with the conjugate leads to receptor-dependent selective down-regulation of the endogenous miRNA levels and up-regulation of validated miRNA targets in U87MG cells. Therefore, the authors reported the effects of GL21.T-222 chimera in inhibiting migration ability and sensitizing the cells to TMZ treatment *in vitro*, and its inhibitory effect on tumor growth *in vivo*. Furthermore, they performed the conjugation of two different anti-miR sequences to GL21.T aptamer (GL21.T-10b-222) enabled to combine the effects of each anti-miR in reducing the intracellular levels of both miR-222 and miR-10b and, in turn, by increasing the specific target protein levels [64]. The same aptamers have been used as carriers for miR-137 and anti-miR-10b molecules to target in combination the GSC population (Axl^+^/PDGFRβ^+^) in a receptor-dependent manner, by adopting a stick-end approach for the non-covalent conjugation. The authors reported that treating cells with the two conjugates in combination inhibits tumorsphere formation and migration, both in U87MG tumorspheres and in patient-derived GSC lines. A combined targeting approach towards GSC population was also reported with Gint4.T-STAT3 AsiC. Particularly, Esposito and colleagues showed that the combined treatment of Gint4.T-STAT3 with GL21.T-10b resulted in a synergistic and drastic inhibition of GSC self-renewal [62]. Moreover, they showed that the aptamer conjugates are transported through an *in vitro* BBB tri-culture model likely by a receptor-dependent mechanism mediated by Axl and PDGFRβ receptors expressed by endothelial cells and pericytes [65]. The ability of aptamer conjugates to selectively target GSC population has been also demonstrated by Affinito and colleagues. They characterized the aptamers 40 L and A40s as high affinity ligands for Eph receptor A2 (EphA2), which is overexpressed in a subset of GSCs, and generate aptamer chimeras by using sticky-end annealing. In particular, they treated GSCs with A40s-miR34c or A40s-anti-miR-10b conjugate showing the modulation of the expression of the corresponding miRNA [51,72].

Noteworthy, aptamer-based approach represents an effective strategy for the targeted delivery of catalytic oligonucleotides as well. Larcher and colleagues developed a type of DNAzymes able to target and inhibit miR-21, termed antimiRzyme RNV541. By employing a previously developed DNA aptamer against TfR, they generated the TfR-RNV541 conjugate enabled to be internalized into U87MG cells and to inhibit the expression of endogenous miR-21 [66].

### Aptamer-nanoparticle complexes

In recent years, NPs for GBM therapy have drawn attention due to their proven advantages with respect to conventional strategies for tumor therapy. These include BBB and blood-brain tumor barrier (BBTB) crossing and delivering of active agents, including NATs, specifically to GBM cells, by exploiting active targeting strategies [73]. Herein, we will specifically focus on the application of Ap-NP complexes in delivering NATs. In the context of GBM, Ap-NP complexes have been developed, to date, for siRNA delivery and their main features have been summarized in Table 1.

The first example of an Ap-NP complex designed for siRNA delivery was presented by Sun and colleagues. The authors developed cationic liposomes loaded with paclitaxel and survivin siRNA, endowing the nanovectors with dual targeting properties. By exploiting 1-Ethyl-3-(3-dimethylaminopropyl)carbodiimide (EDC) chemistry, they functionalized the liposomes’ surface with two ligands. The first ligand, Angiopep-2, enhances the BBB and BBTB crossing [74]. The second ligand, the DNA Aptamer 15, specifically binds to CD133, which is a marker of GSCs. The Ap-NP complex exploits the synergistic action of paclitaxel and survivin siRNA, indeed the siRNA decreases survivin mRNA level in U251-CD133^+^ cells, inhibiting survivin effect as anti-apoptotic protein and, consequently, enhancing the efficacy of paclitaxel [67]. Intriguingly, another strategy was developed by Zhou and colleagues to deliver specific siRNA against survivin. The authors exploited DNA tetrahedron nanomaterial to deliver this siRNA for targeting GBM cells through the DNA aptamer AS1411 [68]. The DNA tetrahedron is a 3D structure based on DNA material and formed by four single-stranded DNA. It was engineered to attach the siRNA and the aptamer to two of its strands. The AS1411 aptamer specifically binds to nucleolin, highly expressed in GBM, enabling targeted delivery to U87MG cells. Indeed, using fluorescence microscopy, it has been demonstrated that Ap-NPs were preferentially internalized by GBM cells compared to human umbilical vascular endothelial cells (HUVECs). Similarly to the previous findings, the developed nanovector leads to a reduction of survivin expression, consequently triggering the apoptosis of GBM cells [68].

Fei and colleagues developed an Ap-NP complex by covalently attaching the chimera Gint4.T-siHDGF to the surface of mesoporous silica NPs (MSNs), loaded with TMZ. As described in the previous paragraph, the chimera is able to specifically recognize GBM cells and interfere with GBM proliferation, migration and invasion. Moreover, it serves as a capping agent and it is removed under the acidic environment of lysosome, triggering the controlled release of TMZ from the inside of the NPs. Thus, the co-delivery of TMZ and siRNA enhances the therapeutic efficacy of the nanovector against GBM. The incorporation of siHDGF into the chimera enhances its stability compared to naked siRNA, which is susceptible to degradation by nucleases. When conjugated to the surface of NPs and administered as TMSN@siHDGF-Gint4.T in GBM-bearing mice, the siHDGF adopts a dense and oriented configuration that prevents nuclease attacks, enhancing its *in vivo* stability and prolonging blood circulation time [63]. Recently, MnO_2_ NPs have also been exploited for the delivery of siRNAs. The authors designed an Ap-NP complex by employing ultrasonic fusion to modify hollow H-MnO₂ NPs with a camouflage membrane derived from brain metastatic B16F10 cells, which was further engineered with TfR aptamers. This membrane serves as the outer shell of the Ap-NP complex, enhancing its ability to traverse BBB effectively. Indeed, the membrane endows the Ap-NP complex with biomimetic properties, mimicking endogenous cells. Moreover, the ability to cross BBB is further enhanced by the active targeting properties of the aptamer. The NPs were loaded with KKGKGQQ-tetraphenylethene (Pep-TPE), which self-aggregates in the presence of transglutaminase 2 (TG2) biomarker, triggering fluorescence for *in situ* imaging of GBM. Additionally, the nanovector encapsulates siRNA that silences TG2 overexpression, which has been closely associated with cancer progression [57].

To develop Ap-NP complex capable of specifically targeting GBM cells, Gu and colleagues designed composite NPs (CNPs) modified with the DNA aptamer SL1, which specifically targets c-Met, highly expressed on GBM cells. Particularly, the core of the NPs consists of MSNs loaded with a siRNA against the cleavage factor Im 25 (CFIm25). It is a crucial component of the CFIm complex, playing a key role in regulating the length of the mRNA 3′ untranslated regions (3′UTRs), thus impacting on cell function. To protect the siRNA from premature degradation, the MSNs are encapsulated within liposomes, which act as a sealing layer for the NP pores, and the surface has been modified with the aptamer SL1, to enhance the targeting efficiency. This Ap-NP complex showed the ability to silence CFIm25, leading to changes in the post-transcriptional regulation. Once internalized by GBM cells, the siRNA is released from the CNPs and CFIm25 is silenced, leading to significant shortening of the 3′UTRs of several tumor suppressor genes (*e.g., TP53, PTEN*, and *RB1*), increasing their mRNA levels. *In vitro*, the Ap-NP complex significantly inhibited cell proliferation, promoted apoptosis, and reduced the migration and invasive ability of GBM cells. Moreover, these effects have been confirmed *in vivo* experiments, showing that the treatment with the nanovector prolonged survival in GBM-bearing mice [69].

### Aptamers *versus* nanoparticles: advantages and disadvantages as delivery systems

Aptamers offer numerous advantages for brain tumors thanks to unique repertoire of special features. Firstly, aptamers have small size and low molecular weight (<20 kDa) allowing an efficient tissue penetration. Their chemical nature offers the advantage of easy and cost-effective synthesis procedure. Indeed, they can be chemically synthesized, which allows rapid productions and versatile chemical modifications for improving their pharmacokinetics and pharmacodynamics [75]. Moreover, their 3D-structure makes them able to recognize a wide range of potential biological targets including ions, drugs, peptides, nucleic acids, proteins, viruses, live cells and tissues, with high affinity and specificity enabling selective binding of tumor cells and limiting the side effect on the healthy cells [23]. Altogether, these features make aptamers able to satisfy one of the fundamental requirement for GBM therapy, *i.e.* the BBB crossing, as demonstrated by several potential therapeutic strategies developed over the years, as described in previous paragraphs. Another very important advantage of using aptamers as delivery system is the lack of immunogenicity and toxicity that permits a safe administration even of repeated doses [76]. However, in order to improve the translation potential of aptamer-NAT conjugates some hurdles need to be addressed. Although the nature of aptamers does not affect their mechanism of action, DNA and RNA aptamers show distinct advantages in terms of chemical and conformation stability and 3D-structure [77]. Particularly, RNA aptamers show a high conformation stability due to the strong RNA-RNA interaction [78], but they are easily degraded by nuclease digestion when used *in vivo*, which contributes to their limited translation potential. Another issue is the low amount of chimera able to reach the cytoplasm and act on its specific target. After internalization into the target cell upon endocytosis, only 1 % of the aptamer conjugate escapes the endosomal compartment reaching the appropriate subcellular region to exert its targeting function [75]. Thus, the scarce ability of aptamers to enable effective endosomal escape of NATs might result in reduced therapeutic effectiveness and increased dosage. Several strategies based on NPs have been devised to enhance the endosomal release of NATs. Among them, polymer nanoparticles, which are susceptible to pH variations, undergo alterations in their structure when exposed to the acidic conditions inside the endosome, so allowing the endosomal escape. Other approaches consist in engineering NPs with cytosol-penetrating polypeptides, which are used to form membrane pores or alternatively, with ionizable phospholipids, which disrupt the membrane of endosome by forming a cone shape at low pH conditions [79]. Other than facilitate the endosomal escape of NATs, NPs have some additional advantages as delivery systems. Based on their composition, NPs can be classified in five major groups (lipid NPs, polymeric NPs, peptide NPs, biomimetic NPs, and inorganic NPs) and each group has different advantages and disadvantages, as widely described in [80]. Overall, NPs preserve the stability of NATs, protecting them from nuclease degradation, ensuring they reach their target cells. Furthermore, incorporating NATs into NPs can improve their pharmacokinetic properties, such as in prolonged blood circulation *in vivo*. Different therapeutic agents can also be loaded simultaneously to NPs for combination therapy, allowing to interfere with different signaling pathway crucial for GBM progression [81].

The enhanced permeability and retention (EPR) effect helps NPs to accumulate passively in cancer tissues, exploiting the leaky vasculature and scarce lymphatic drainage in cancer. However, EPR effect is not exclusive to cancer tissues, and can determine the accumulation of NPs in normal tissues, causing non-specific distribution and toxicity [82]. NP-based delivery is also limited by immunogenicity, susceptibility to aggregation in biological fluids, leading to decreased efficacy and potential toxicity [38]. Another disadvantage to overcome in using NPs as delivery system in GBM therapy is the lack of ability to cross the BBB. Only few types of NPs are able to cross the BBB through passive diffusion, such as small lipophilic cationic NPs and gold NPs. Nevertheless, passive diffusion alone may not be sufficient for effective targeting [83]. A possible strategy to overcome these challenges is the conjugation of NPs with specific ligands, such as aptamers. As mentioned above, several aptamers able to cross the BBB and specifically bind GBM tissue have been designed, allowing the development of more suitable therapeutic approaches for GBM treatment based on the combination of these two delivery systems.

## Conclusions and perspectives

To date, the main limits in the development of innovative and effective GBM treatment are represented by the BBB penetration and poor cell targeting that alter the delivery of therapeutic agents to the tumor site, leading to severe off-target effects. Aptamers could represent a promising opportunity to overcome these obstacles. Thanks to their easily modifiable chemical nature, different aptamer-based conjugates have been developed for the targeted delivery of therapeutic agents. Thus far, aptamers have been functionalized to make them capable of further conjugation with different molecules, including chemotherapy agents. The application of aptamers as specific drug delivery system represents a significant advantage for more effective and precise cancer treatment approaches. Thanks to their ability to cross the BBB, reach specifically GBM cells and release their cargo, aptamers enable to overcome issues typical of traditional drugs, such as high toxicity both on cancer and healthy cells and lack of active targeting, allowing a concrete reduction of dosage and side effects. These features make aptamer promising candidates for drug delivery in GBM compared to conventional therapy [38]. However, despite the above-mentioned advantages, some limitations must be considered. The most important one is their instability due to nuclease degradation. A possible strategy to protect aptamer from nuclease degradation is the introduction of chemical modifications within their sequence. The most common are: phosphate linkage modifications; the introduction of a 3′- or 5′- cap with several molecules (*e.g.*, biotin–streptavidin, inverted thymidine, polyethylene glycol or cholesterol); modifications of bases in the position 5 of pyrimidine and position 8 of purine; modification at the 2′ and 4′ positions of the ribose (2′-F, 2′-NH_2_, 2′-O-CH_3_, 4′-S, or locked nucleic acids). Alternative strategies for stabilizing aptamers include the generation of “mirror image” aptamers known as “spiegelmers”, circular aptamers and slow off-rate modified aptamers (SOMAmers) [84]. The conjugation of aptamers to NPs, forming Ap-NP complexes, represents another strategy to prevent enzymatic degradation of aptamers [85]. In addition, NAT-loaded Ap-NP can protect the cargo, addressing challenges generally faced by naked oligonucleotide such as inefficient cellular transfection, short circulation time, endogenous degradation and off-target effects [86]. Thus, even if the direct aptamer-NAT conjugation can be considered a promising delivery strategy, the Ap-NP complexes can offer further advantages. Taking advantage of chemical versatility of aptamers, several methods have been developed to use them for functionalizing NPs. Aptamers can be conjugated with several kinds of NPs such as polymeric and lipid NPs, dendrimers, metallic and magnetic NPs, DNA nanomaterials, and biomimetic NPs [70,87]. Noteworthy, the produced Ap-NP complexes, show only a slight increase in NP size, lower than 100 nm, that has been shown to be suitable for an efficient BBB crossing and the loading capacity of the targeted receptors [88]. Therefore, the advantages associated to the incorporation of NATs into Ap-NP complexes, as delivery system, are: i) enhanced pharmacokinetic properties, including a prolonged circulation half-life*;* ii) NAT protection from nuclease degradation; iii) enhanced cellular uptake of NATs due to their small size; iv) ability to incorporate more therapeutic agents compared to aptamer alone [70,81,85,89].

Overall, the BBB crossing ability, specific GBM targeting and an increased aptamer and NAT stability could improve therapeutic efficacy, making aptamers alone or Ap-NP complexes promising tools for GBM therapy.

## Funding information

This work has been partially conducted under the National Plan for Complementary Investments to the NRRP, project “D34H—Digital Driven Diagnostics, prognostics and therapeutics for sustainable Health care” (project code: PNC0000001), Spoke 3, funded by the Italian Ministry of University and Research. This work was supported by Associazione Italiana per la Ricerca sul Cancro, AIRC (IG 2021 26515) to LRV.

## CRediT authorship contribution statement

**Giorgia Castellani:** Writing – review & editing, Writing – original draft, Conceptualization. **Mariachiara Buccarelli:** Writing – review & editing, Writing – original draft, Conceptualization. **Enza Cece:** Writing – original draft. **Martina Offi:** Writing – review & editing. **Lucia Ricci-Vitiani:** Writing – review & editing.

## Declaration of competing interest

The authors declare that they have no known competing financial interests or personal relationships that could have appeared to influence the work reported in this paper.
